# Intrinsic motivation in cognitive architecture: intellectual curiosity originated from pattern discovery

**DOI:** 10.3389/frai.2024.1397860

**Published:** 2024-10-17

**Authors:** Kazuma Nagashima, Junya Morita, Yugo Takeuchi

**Affiliations:** ^1^Department of Information Science and Technology, Graduate School of Science and Technology, Shizuoka University, Hamamatsu, Japan; ^2^Department of Behavior Informatics, Faculty of Informatics, Shizuoka University, Hamamatsu, Japan

**Keywords:** cognitive modeling, ACT-R, intrinsic motivation, intellectual curiosity, pattern discovery

## Abstract

Studies on reinforcement learning have developed the representation of curiosity, which is a type of intrinsic motivation that leads to high performance in a certain type of tasks. However, these studies have not thoroughly examined the internal cognitive mechanisms leading to this performance. In contrast to this previous framework, we propose a mechanism of intrinsic motivation focused on pattern discovery from the perspective of human cognition. This study deals with intellectual curiosity as a type of intrinsic motivation, which finds novel compressible patterns in the data. We represented the process of continuation and boredom of tasks driven by intellectual curiosity using “pattern matching,” “utility,” and “production compilation,” which are general functions of the adaptive control of thought-rational (ACT-R) architecture. We implemented three ACT-R models with different levels of thinking to navigate multiple mazes of different sizes in simulations, manipulating the intensity of intellectual curiosity. The results indicate that intellectual curiosity negatively affects task completion rates in models with lower levels of thinking, while positively impacting models with higher levels of thinking. In addition, comparisons with a model developed by a conventional framework of reinforcement learning (intrinsic curiosity module: ICM) indicate the advantage of representing the agent's intention toward a goal in the proposed mechanism. In summary, the reported models, developed using functions linked to a general cognitive architecture, can contribute to our understanding of intrinsic motivation within the broader context of human innovation driven by pattern discovery.

## 1 Introduction

According to Baron-Cohen (2020), human evolution and the development of civilization are associated with “systematizing mechanisms,” which are achieved by discovering, combining, and using patterns of cause-and-effect relationships in an environment. He also stated that the ability of humans to think systematically has evolved by using the “if-and-then” logic to combine patterns, resulting in inventions and innovations that lead to our modern society.

Several studies have reported that such an ability of pattern discovery is associated with fun, a personal feeling leading to intrinsic motivation (Caillois, 1958; Csikszentmihalyi, 1990; Huizinga, 1939; Koster, 2013). The other researchers (Aubret et al., 2019; Schmidhuber, 2010) have also explored the computational realization of intrinsic motivation employing the framework of reinforcement learning (Sutton and Barto, 1998). However, these studies have not explored the link between intrinsic motivation and primitive cognitive functions related to pattern discovery. Therefore, further analysis of the computational mechanisms of intrinsic motivation in terms of agents' internal processing is needed.

The aforementioned problem can be addressed by using a cognitive architecture that integrates the basic cognitive functions involved in various tasks. Despite the existence of several cognitive architectures, the architectural differences have been reduced over the years and integrated into a common structure (Laird et al., 2017). The representative architecture adopting such a structure is adaptive control of thought-rational (ACT-R), developed by Anderson (2007). According to Kotseruba and Tsotsos (2020)'s comprehensive review of the topic, ACT-R is one of the most widely used cognitive architectures, including a greater number of features compared to the other architectures.

In this study, we propose a mechanism of intrinsic motivation based on pattern discovery by integrating primitive cognitive functions of ACT-R. The main advantage of the proposed approach is its interpretability. Based on commonly used building blocks in the architecture, our proposed mechanism can provide a foundation for understanding intrinsic motivation from the perspective of human cognition. Furthermore, this study presents a simulation experiment to explore the conditions of stimulating intrinsic motivation and the learning process driven by stimulated intellectual curiosity. Our analysis confirmed that the proposed mechanism can represent the role of intellectual motivation in human learning at diverse levels of thinking and task difficulty. Additionally, we examined the relationship between the proposed mechanism and an existing mechanism of intrinsic motivation based on reinforcement learning.

The remainder of this paper is organized as follows. Section 2 summarizes the existing studies related to this concept. Section 3 introduces the proposed mechanism, which is developed based on pattern discovery. The effectiveness of the mechanism is discussed based on simulations in Section 4. Finally, Section 5 summarizes the findings and indicates directions for future investigations.

## 2 Related works

The objective of this study is to represent a mechanism of intrinsic motivation based on the discovery of patterns. This section focuses on three directions of previous studies, namely, human curiosity, machine curiosity, and cognitive models with cognitive architectures.

### 2.1 Human curiosity

Numerous studies have attempted to systematize intrinsic motivation as a driving factor to continue activities in a wide range of fields, including education, entertainment, healthcare, sports, and work. For instance, Malone (1981), who tried to systematize this concept in entertainment fields, categorized intrinsic motivation into three types, namely, “challenge,” which originates from goals of appropriate difficulty; “fantasy,” which leads to the imagination of unrealistic experiences; and “curiosity,” which is stimulated by a surprising, interesting, or fun activity. Here, curiosity is related to the discussion presented in Section 1 that pattern discovery accompanying the feeling of fun has led to human innovations. However, we believe that the first type of intrinsic motivation, challenge, is inseparable from curiosity. Rather than treating those as independent factors, we assume that curiosity is a mechanism of intrinsic motivation, stimulated by the appropriate difficulty (challenge) of a task.

The above assumption is supported by several authors who reported the relationship between the levels of task difficulty, the preferred level of thinking, and intrinsic motivation. The theory behind this is referred to as the optimal level of intrinsic motivation (Csikszentmihalyi, 1990; Yerkes and Dodson, 1908). According to this theory, intrinsic motivation is effectively stimulated when the task difficulty level matches the preferred level of thinking of a person. Furthermore, the level of thinking can be located on an axis with at least two levels. These include a shallow automatic level without careful thinking (fast process) and a deep deliberative level that requires time to carefully think (slow process) (Brooks, 1986; Evans, 2003; Kahneman, 2011).

### 2.2 Machine curiosity

Based on the aforementioned discussion, we assumed a close relationship between curiosity and the feeling of fun involved in the discovery of patterns. This relationship was computationally theorized by Schmidhuber (2010), wherein the discovery of patterns is defined as identifying and compressing recurring canonical patterns in data. Schmidhuber also related compressing data or obtaining compressible data to fun by assuming that the agent aims to maximize fun as a reward. This idea was based on the prediction error theory (Friston, 2010), which considers curiosity to be caused by the difference between prior predictions and the current situation (Bayesian surprise). In Schmidhuber's theory, prediction implies applying already compressed data; here, surprise occurs when identifying a pattern that can be newly compressed.

Schmidhuber's proposal can be discussed as an extension of conventional reinforcement learning. Typically, agents in reinforcement learning receive rewards from the environment and intend to maximize them over time. Sutton and Barto (1998) distinguished the boundaries between the agent and the environment from the physical boundaries between the body and the environment. Based on this idea, Singh et al. (2005) proposed a framework of intrinsically motivated reinforcement learning (IMRL), which divides the environment into external and internal segments. In contrast to conventional reinforcement learning, which directly receives a reward from the external environment, rewards in IMRL are determined depending on the state of the internal environment, such as stimulating curiosity for an unexpected response.

Since the proposal of IMRL, the framework of reinforcement learning has significantly progressed by integrating deep learning techniques. The preliminary framework was referred to as Deep Q Network (DQN) (Mnih et al., 2015), which combined Q learning with a convolutional neural network (CNN). Subsequently, several researchers introduced the concept of intrinsic motivation in deep reinforcement learning. For instance, Bellemare et al. (2016) developed count-based exploration methods, wherein visit counts were used to guide an agent's behavior toward reducing uncertainty. In their research, calculating prediction errors as internal rewards led the agents to search for novel states and ultimately outperformed DQN. Following this idea, Pathak et al. (2017) proposed the intrinsic curiosity module (ICM), which regarded the difference between the predicted state of an agent and the situation obtained from the pixel information on the screen as curiosity. Herein, ICM was integrated with the asynchronous actor-critic model (A3C) (Mnih et al., 2016). Based on this method, Burda et al. (2018) implemented an approach to explore the environment using only internal rewards regarding curiosity. Moreover, Burda et al. (2019) proposed a method named random network distillation, which made it possible to learn tasks that were difficult to accomplish with the previous methods.

### 2.3 Cognitive models with cognitive architectures

Although the aforementioned studies successfully represented curiosity in reinforcement learning, their integration with cognitive functions has not been sufficiently explored. As explained in Section 2.1, curiosity is associated with the discovery of patterns. Therefore, the computational representation should include basic human cognitive functions behind pattern discovery; this can be achieved using ACT-R. The subsequent section explains the representation of individual cognitive functions in ACT-R and the type of learning realized by combining cognitive functions. Herein, we predominantly focus on the cognitive functions of ACT-R involved in this study. Further information on ACT-R can be obtained from Anderson (2007), the ACT-R manual (Bothell, 2020), and other reviews (Ritter et al., 2019).

#### 2.3.1 Structure of ACT-R modules

**Figure 1:**
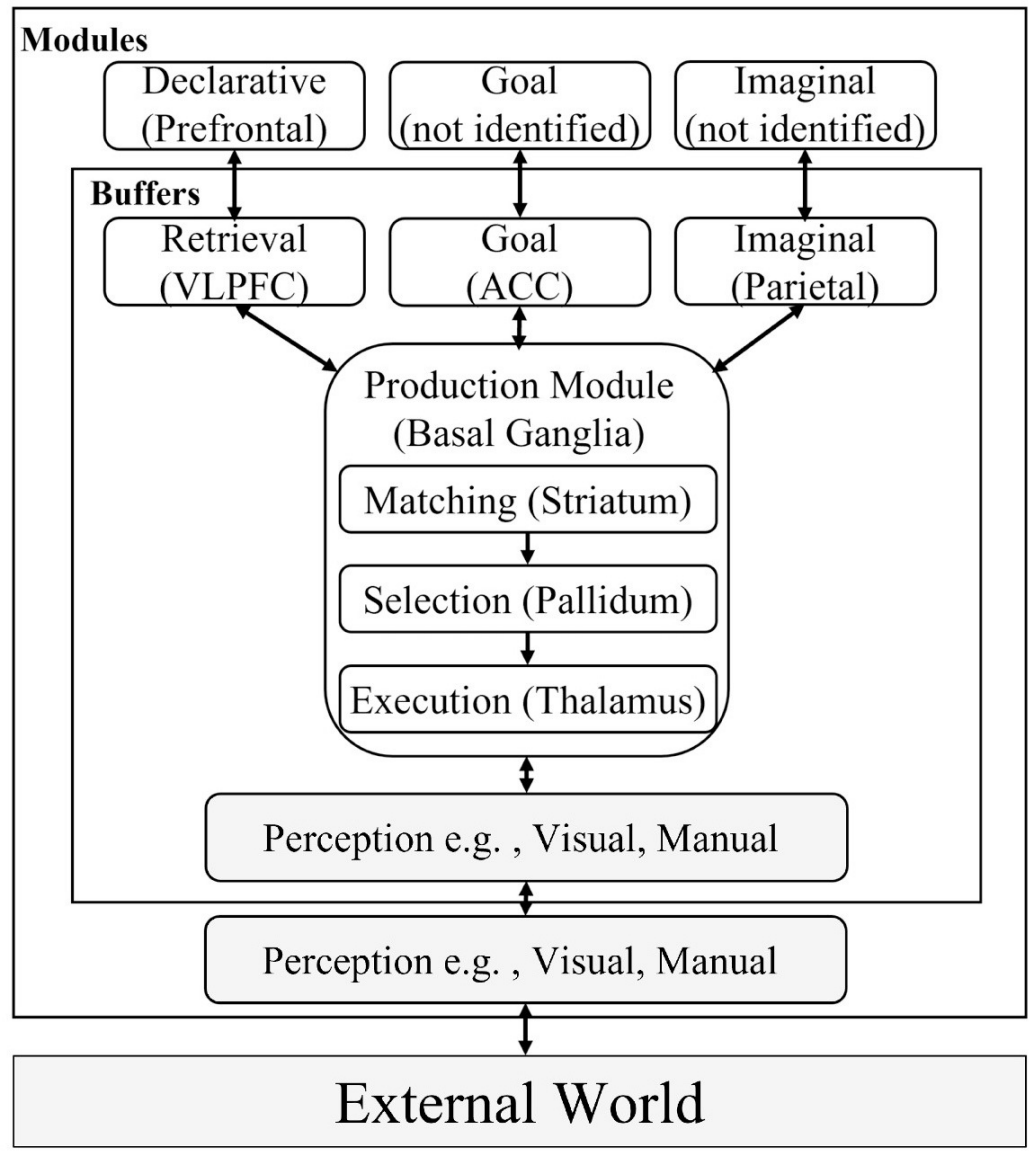
Overview of the adaptive control of thought-rational (ACT-R) modules. Modules excluded in this study are grayed out. This figure is created with reference to Anderson et al. (2004) and Ritter et al. (2019). VLPFC, ventrolateral prefrontal cortex; ACC, anterior cingulate cortex; DLPFC, Dorsolateral prefrontal cortex; PPC, posterior parietal cortex.

ACT-R comprises modules corresponding to brain regions, as indicated in Figure 1. The mapping between the modules and regions has been discussed based on neuroscientific findings (Stocco et al., 2021). The principal assumption of this structure is that one module takes responsibility for a set of functions. For instance, the declarative module comprises functions for storing experience and knowledge, the imaginal module contains functions to create new knowledge by combining multiple internal representations, and the function of the goal module is to maintain the current status of tasks to manage the process of the model. The state of each module at each time point (e.g., the declarative knowledge being recalled and the state of the current goal) is expressed using a symbol referred to as a *chunk*, which is stored in a buffer for each module. The chunks stored in the buffer are evaluated using a type of procedural knowledge, called “productions,” comprising IF (conditions) and THEN (actions) clauses in the production module. The productions transmit chunks describing commands to modules as actions, such as searching for knowledge that satisfies the conditions and updating the current state of the task.

Therefore, the declarative and production modules in ACT-R contain different types of knowledge. The retrieval cost of declarative knowledge (chunks) in the declarative module is greater than that of procedural knowledge (productions) in the production module. The cost in ACT-R corresponds to the processing time, which simulates human reaction times (van der Velde et al., 2022). A single production can be executed in 50 ms, whereas the retrieval of declarative knowledge requires longer as various factors are involved. Moreover, declarative knowledge is not automatically retrieved from the goal module or the external environment as it is always used by applying two productions; one for retrieving declarative knowledge and the other for applying the retrieved knowledge to change the states of buffers (e.g., goal or perceived external environment).

**Figure 2:**
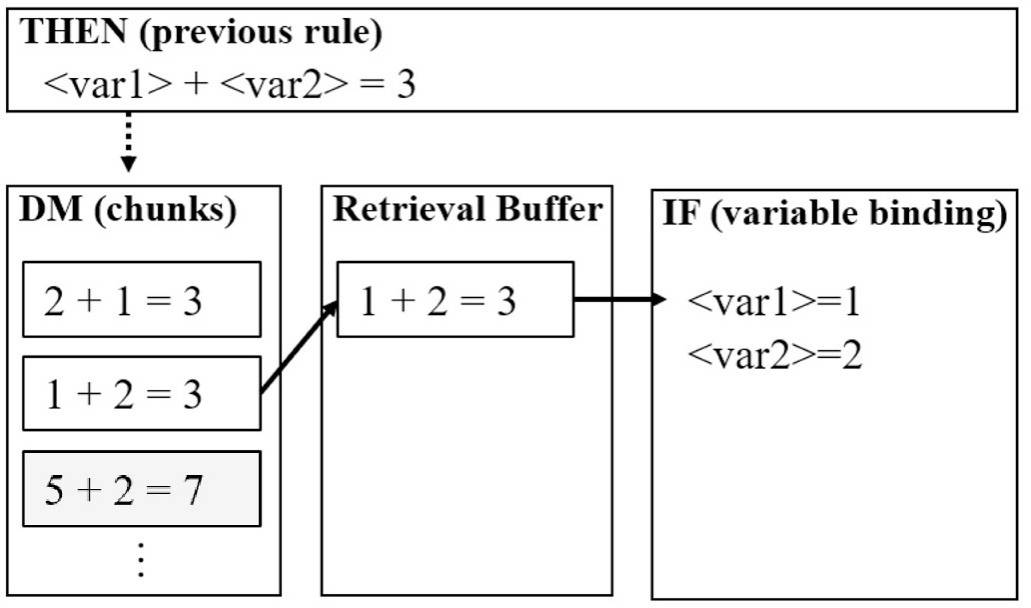
Simple example of pattern matching in the adaptive control of thought-rational (ACT-R) architecture. This example illustrates the flow of a declarative knowledge query to the declarative module (DM) in the THEN clause of the previous production. The variables are bound in the IF clause of the subsequent production.

Biologically, the ACT-R theory assumes that the two types of knowledge are connected through the cortico-basal ganglia loop. As depicted in Figure 1, the productions are assumed to be executed in the basal ganglia; however, the ones used for retrieving declarative knowledge require the prefrontal cortex as well. Figure 2 illustrates a simple example of retrieving and using declarative knowledge through two productions. In the figure, variables “var1” and “var2” in the productions are bound to numerical values, such as 1 and 2, stored in the declarative module. This mechanism is referred to as “pattern matching” and is assumed to be executed intentionally in the prefrontal cortex [particularly in the ventrolateral prefrontal cortex (VLPFC) indicated in Figure 1]. Therefore, we considered the pattern matching between the current situation (buffers) and knowledge in the declarative module as the criterion for distinguishing the aforementioned levels of thinking (Section 2.1). In this framework, the shallow level of thinking involved fewer pattern-matching scenarios than the deliberative level of thinking.

#### 2.3.2 Learning in ACT-R

The existence of pattern matching also makes a distinction between two types of learning in ACT-R: learning with pattern matching and learning without pattern matching. The latter type uses “utility learning,” which corresponds to reinforcement learning. Specifically, it changes the selection probability of productions that conflict with each other by receiving rewards from the environment. Many studies have used this type of learning in ACT-R modeling (Anderson et al., 1993; Balaji et al., 2023; Ceballos et al., 2020; Xu and Stocco, 2021). For example, Fu and Anderson (2006) developed a model to solve the repeated maze task by applying procedural knowledge representing up-down and left-right movements. The model received positive rewards for actions that led to the achievement of the current goal and negative rewards for actions that failed to achieve the goal. As a result of their simulation, the model was able to learn optimal behavior in the maze search by repeating the rewarding trials.

The other type of learning in ACT-R involves pattern matching to retrieve chunks in the declarative module, which is called instance-based learning (IBL) (Gonzalez et al., 2003). This framework accumulates past problem-solving instances in the declarative module and uses it for future task trials. Several studies show that IBL outperforms conventional utility learning. Relating to this method, Reitter and Lebiere (2010) constructed a model to solve maze like Fu and Anderson (2006), but unlike them, by combining path-finding with backtracking and instance-based inference. In their model, location information of the maze was represented as declarative knowledge to construct a topological map (graph-like structure representing geological locations). In addition to conventional knowledge-search algorithms (e.g., depth-first search), an instance-based inference was applied by using stored maze-solving experience in the declarative module. By conducting simulations using the strategies of maze search, they demonstrated the advantage of this memory-based search.

Furthermore, ACT-R contains another learning function that uses the two aforementioned functions. This function is the “compilation,” which combines two productions into a single production (Taatgen and Lee, 2003). During the task execution, this function integrates a repeatedly selected series of productions and reduces the number of productions used in the task as learning progresses. Typically, the target series of compilation involves pattern matching to retrieve declarative knowledge (Figure 2). The function replaces the variables present in the production with instantiated values in the declarative knowledge. Additionally, the conflicting conditions for pre-compiled and post-compiled productions are resolved using the utility learning. The post-compiled production inherits higher utility from those associated with the two pre-compiled productions. Furthermore, the utility of post-compiled production increases with the compilation of the same series of productions. This process increases the probability of selecting a post-compiled production to represent a routine and automatic operation (procedural knowledge) in a task.

#### 2.3.3 Emotion in ACT-R

The subject of the present study, motivation, is considered part of the emotional or affective phenomena in the recently emerging field of affective science.^1^ In this field, researchers have repeatedly pointed to the relations between emotions, cognition, and body (Barrett, 2017; Damasio, 2003; LeDoux and Pine, 2016), underscoring the importance of incorporating emotional and physiological responses into cognitive models.

Following these trends in affective science, several researchers have constructed ACT-R models that represent the interactions between cognition, emotion, and the body. For example, van Vugt and van der Velde (2018) constructed a model explaining depression based on the proportion of memories accompanied by emotional moods. Similarly, Juvina et al. (2018) considered the relationship between emotional memories and reward functions. In addition to these links between emotion and cognition, researchers have included psychophysiological factors such as fatigue (Atashfeshan and Razavi, 2017; Gunzelmann et al., 2009) and stress (Dancy et al., 2015) in ACT-R. Based on these models of emotions, several ACT-R models of motivations have been developed (Nishikawa et al., 2022; Nagashima et al., 2022; Yang and Stocco, 2024). Furthermore, in recent discussions on the common cognitive model, Rosenbloom et al. (2024) proposed an architecture including metacognitive modules to represent interactions between cognition and emotion.

However, to implement such emotional processes, all the aforementioned studies developed novel modules or functions of ACT-R. By contrast, the current study aims to model intrinsic motivation using the existing built-in functions of ACT-R. While we recognize the importance of developing new modules to create a neurally faithful structure, we believe that, in line with the philosophy of cognitive architecture (Anderson et al., 2004), it is preferable to represent various cognitive processes by integrating a small set of core functions.

## 3 Mechanism of intellectual curiosity based on pattern discovery

This section proposes a mechanism of intrinsic motivation. Before presenting details of the mechanism, the basic idea behind our proposal is introduced.

### 3.1 Basic idea

The mechanism proposed here focuses on intellectual curiosity among the types of intrinsic motivation. We used the modifier “intellectual” based on the discussion reported by Malone (1981). According to him, the curiosity derived from higher-cognitive functions is distinguished from that derived from sensory perceptions. He further argued that the former initiates “a desire to bring better form to one's knowledge structures.” This discussion is consistent with the principle of fun discussed by Schmidhuber (2010), who claimed that discovering the compressible structure of data would be beneficial to organize knowledge structures in the agent.

We developed a mechanism of intellectual curiosity by associating ACT-R pattern-matching computation. As explained earlier, pattern matching of ACT-R is a core mechanism for understanding higher-order cognitive processes with the discovery of structures (patterns) that map data (declarative knowledge) to a current situation (buffer states of the module) according to a pattern of variables in the production. This mechanism has been considered essential for achieving cognitive flexibility that adapts changing environments by leveraging existing knowledge in novel forms (Spiro et al., 2012). In fact, Anderson (2007) demonstrated that ACT-R could model human-specific cognitive functions, such as linguistic processing, metacognition, and analogical reasoning by using a certain type of pattern matching.^2^ More importantly, ACT-R pattern matching is involved in the learning mechanisms, as described in Section 2. In the following part of this section, the learning mechanisms of ACT-R are combined into a general framework of intrinsic motivation.

### 3.2 Components of intellectual curiosity

To understand the role of intellectual curiosity in general cognitive processes, we first discuss its decay (boredom) process. Typically, boredom is caused by stimulus saturation and is related to learning processes as suggested by Csikszentmihalyi (1990). According to his theory, boredom occurs when the person extensively learns a particular task and it becomes less challenging. Raffaelli et al. (2018) reviewed research confirming such a process based on subjective and physiological indices, which sometimes showed complex interactions between cognitive and physiological processes. Based on these discussions especially about the relation between learning and boredom, we used the “utility learning” and “production compilation” to represent the decay of intellectual curiosity. Although the general concept of these mechanisms has already been discussed, the subsequent sections focus on the technical details of the modules as ingredients of our integrated mechanism of intellectual curiosity.

#### 3.2.1 Motivation as utility for task continuation

We used utility learning in this study as a mechanism for determining whether a task should be continued or terminated. As mentioned in Section 2.3, the utility learning corresponds to reinforcement learning (Fu and Anderson, 2006). When multiple productions (i.e., the production for task continuation and the production for task termination) match the current situation, the probability of selecting a production can be calculated as


(1) $$\begin{array}{l} P\left(i\right)=\frac{e^{U_{i}/\sqrt{2s}}}{\underset{j}{\sum }e^{U_{j}/\sqrt{2s}}}, \end{array}$$

where *e* denotes the base of the natural logarithm, *s* indicates the parameter that determines the variance of noise according to the logistic distribution, and *j* distinguishes the conflicting productions. Additionally, *U* representing the utility of controlled production can be estimated as


(2) $$\begin{array}{l} U_{i}\left(n\right)=U_{i}\left(n-1\right)+\alpha \left[R_{i}\left(n\right)-U_{i}\left(n-1\right)\right]. \end{array}$$

Here, α represents the learning rate, and *Ri*(*n*) denotes the reward obtained by production *i* at time *n*. In general, rewards occur when a production associated with the goal of the task is executed. Typically, rewards are backpropagated to the productions that are executed before the reward is triggered. Each time a production is rewarded, the utility values of all productions that have been executed since the last update (*n*−1) are updated using Equation 2. In this study, events related to intellectual curiosity and boredom are represented by assigning positive and negative rewards, respectively.

#### 3.2.2 Reduction of pattern matching through production compilation

As mentioned earlier, production compilation compresses productions to reduce frequencies of pattern matching. Therefore, the fun generated by pattern matching (identifying structures in the data) was considered decayed by the compression accompanied with production compilation.

**Figure 3:**
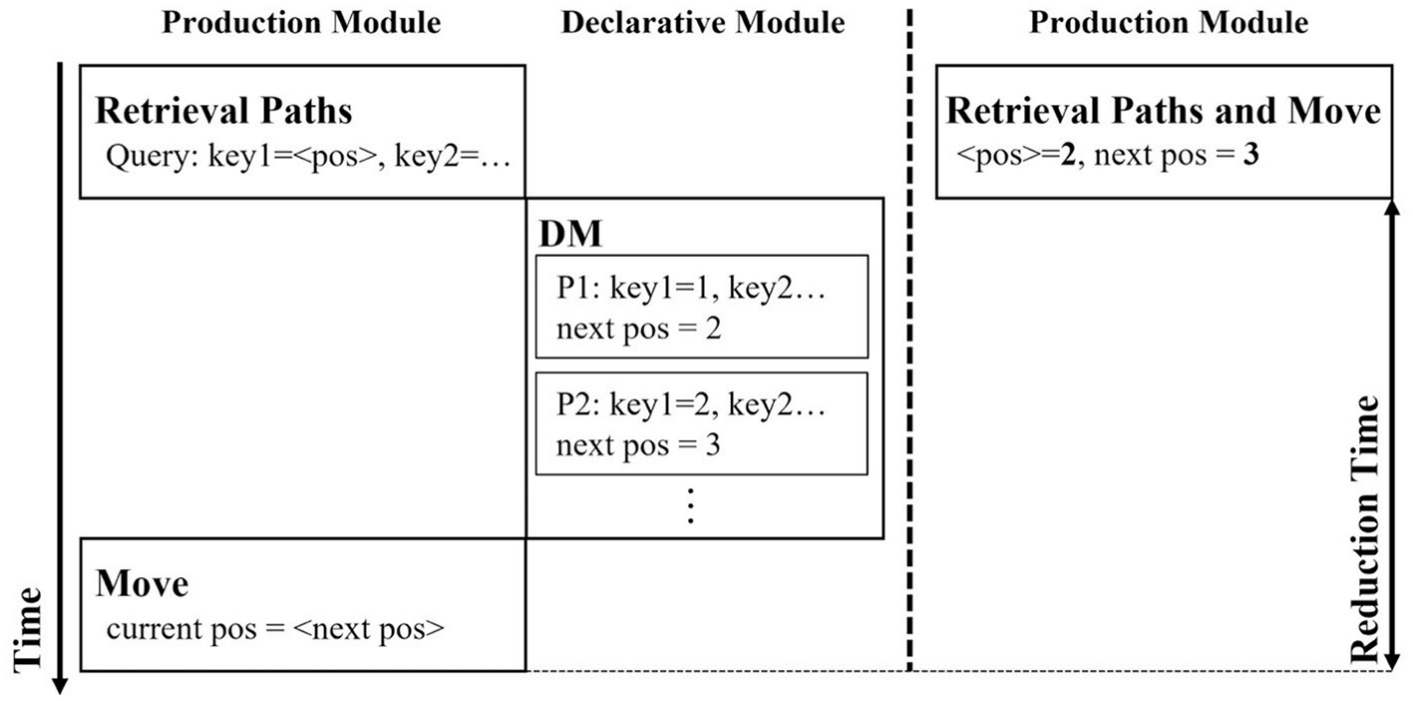
Example illustrating the before and after of learning using the production compilation.

Figure 3 depicts the traces of an ACT-R model in a maze task used in simulations performed in this study. The vertical axis indicates time, and each column indicates an event in a module. The left-hand side trace represents the process of identifying the path from the declarative knowledge using pattern matching. The trace on the right-hand side expresses the search for a path without pattern matching or retrieving paths from the declarative module; in other words, it represents the processing after production compilation.

### 3.3 Integrated mechanism of task continuation based on intellectual curiosity

**Figure 4:**
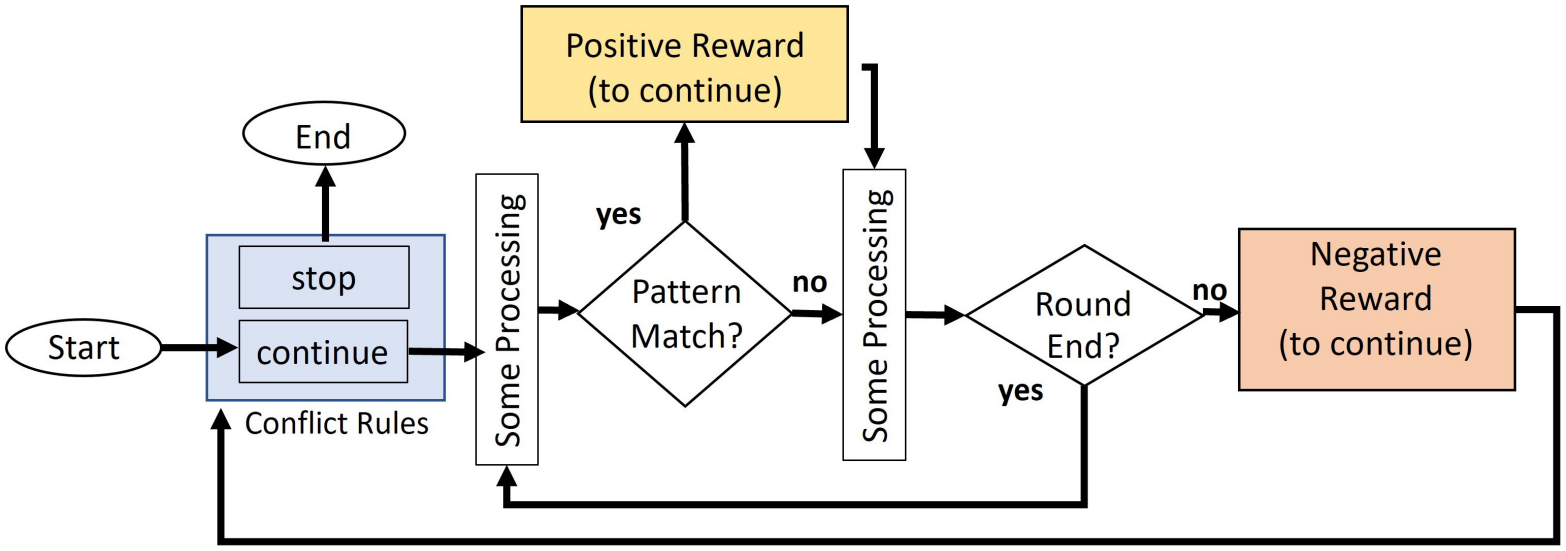
Flowchart of the task continuation model. The model generates positive rewards when pattern matching occurs.

We propose a mechanism for determining the continuation or termination of a task based on intellectual curiosity. Figure 4 illustrates the procedure of task continuation when executing general tasks. At the beginning of each round (unit related to the continuation of a task), the model determines whether to continue or terminate the task based on the conflict resolution between the two productions (*stop* and *continue productions*). The model proceeds with the round by firing various productions, such as searching the map, after deciding to continue the task. When the model encounters a condition that terminates the round, a new round is initiated, and the model again determines whether the task should be continued or terminated.

In the aforementioned process, the assigned initial values of utilities are higher in the continue production than in the stop production. At the beginning of the task, it can be assumed that agents intend to continue the task. The process of experiencing boredom from this initial state can be modeled by assigning a trigger of negative reward to the production recognizing the end of each round.^3^ The utility of the production decreases when a negative reward is generated by the continue production at the end of the round, which in turn increases the firing probability of the stop production.

To deter boredom and continue the task, positive rewards corresponding to “fun” are necessary. This study associates the occurrence of pattern matching with the feeling of fun. We consider that this association is consistent with the definition of fun reported by Schmidhuber (2010) because it involves the discovery of patterns in the environment. However, repeated application of the same production causes habituation (production compression) and increases the opportunity to generate negative rewards to the continue production at the end of a round. In other words, the factor that ensures task continuation in the mechanism is the continued stimulation of intellectual curiosity through the discovery of declarative knowledge (data), which is the target of pattern matching.

## 4 Simulation

We performed simulations to verify the proposed mechanism of intellectual curiosity. This section explains the purpose of the simulations, the employed task, model details, and other settings involved in the simulations. Finally, the obtained results are summarized.^4^

### 4.1 Aims and indicators

To examine the mechanism of intellectual curiosity based on pattern discovery, we address the following questions.

1. What type of environment stimulates intellectual curiosity?
2. How does stimulated intellectual curiosity affect task learning?
3. What is the relationship between the proposed mechanism and the curiosity represented in existing reinforcement learning models?

The first question was answered by distinguishing between *external* and *internal* environments surrounding the model. Here, based on the previous discussion on IMRL (Singh et al., 2005), we adopted the term internal environment to explore the individual differences surrounding and affecting intellectual curiosity. In this context, the external environment was manipulated by varying the complexity (difficulty level) of the learning environment, while the internal environment was defined as the strategy employed by the model to explore the external environment.

Furthermore, we examined how the factors of the internal and external environment affect intellectual curiosity using the indicators

(a) up-time ratio (percentage of time the model was running relative to the time limit of one run); and
(b) number of rounds (frequency of firing the task continuation production, depicted in Figure 4).

These indicators represent the extent to which the model engaged with the task. By definition, if the model obtains strong motivation, these indicators are assumed to be increased. We explored the internal and external environments that fostered this effect. As an internal environment, we manipulated the depth of thinking when searching external environments. According to the discussion presented in Section 2.1, this factor is expected to affect the effect of intrinsic motivation of the model via the interactions with the difficulty level of the external environment.

To answer the second question, the effect of stimulated intellectual curiosity on learning in the task was examined using the indicators

(c) entropy (variety of behavior patterns in the environment search);
(d) goal rate (the goal achievement rate); and
(e) the number of newly generated productions (frequency of occurrence of production compilation).

These indicators quantify the effect of intellectual curiosity on three aspects, namely, the behavior pattern (c), learning outcome (d), and internal states (e). We assumed that these indicators would increase with higher intellectual curiosity. In other words, the higher the motivation, the more opportunities the model has to explore the map. Moreover, as the model is extensively exploring the map, entropy (c) and the goal rates (d) increase while the model discovers more patterns in the external environment (e).

The complexity of model behavior (c) was computed as the entropy normalized for the frequency of occurrence of states of the task as follows:


(3) $$\begin{array}{l} Hr=\frac{-\underset{i\in \text{n}}{\sum }p\left(x_{i}\right)\log p\left(x_{i}\right)}{\log n} \end{array}$$

Here, *x*_*i*_ denotes a particular state in an environment, and *n* represents the total number of states in an environment. This index increased when the model extensively explored the environment and the value decreased during local behaviors.

Finally, to address the last question, we used these indicators to examine whether the proposed behavior of curiosity was consistent with the previous models of curiosity. Among several models, we focused on the ICM model (Pathak et al., 2017) as the representative mechanism of deep reinforcement learning with curiosity and compared it with the ACT-R models with various internal and external environments.

### 4.2 Task: manipulation of the external environment

**Figure 5:**
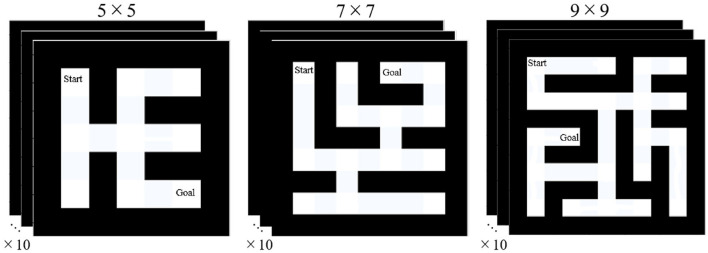
Manipulation of external environments.

Based on the previous reports (Fu and Anderson, 2006; Reitter and Lebiere, 2010) on ACT-R explained in Section 2.3.2, we adopted the task of searching mazes. To systematically manipulate the difficulty level of the task, we applied a maze generation algorithm^5^ to grids of sizes 5 × 5, 7 × 7, and 9 × 9, with 10 different maps prepared for each size; Figure 5 depicts an example of the maps. As indicated in the figure, the created mazes are loop-less structures with the starting location at the top-leftmost corner, and the goal location at the corner where the maximum number of corner points is traversed from the start point. In other words, two corner points with the highest number of hops were selected as the start and goal locations. The difficulty level of this task corresponded to the size of the maps. As described in Section 2.2, we assumed that an appropriate level of difficulty stimulates intrinsic motivation. Therefore, the factors that stimulate the proposed intellectual curiosity were examined by comparing different sizes of the external environments.

This task was implemented in ACT-R using a simplified method to obtain stable results over numerous runs. Rather than presenting a visual representation of the map to the model, we included chunks representing the structure of the map in the declarative module of the model.^6^ In other words, the task corresponded to a situation where the model performed path planning without actually moving the body with respect to the topologically represented declarative knowledge of the environment.

The topological map provided to the model comprised chunks representing nodes (corner points) and paths (connections between the corner points) of the maze. When the task was executed, the model stored a node chunk in the goal module that indicated the currently focused corner point. From this state of the goal module, the model attempted to discover the chunk of paths stored in the declarative modules by matching them with patterns of variables embedded in the productions. When the chunk containing the current node was retrieved from the declarative module, the other node associated with the corresponding path chunk was newly stored in the goal module. This process was repeated until the model reached the goal point or the designated time was elapsed.

### 4.3 Search strategy: manipulation of the internal environment

To examine the internal environment that stimulates intellectual curiosity, we manipulated the strategy of exploring the external environment in terms of different levels of thinking (Brooks, 1986; Evans, 2003; Kahneman, 2011). As explained in Section 2.1, human mental activities are traditionally divided into at least two levels despite a continuous debate on the simple separation. This study follows the discussion reported by Conway-Smith and West (2022), suggesting that individual mental process is characterized by a spectrum between the fast automatic and slow deliberate processes. According to them, the levels in this spectrum can determine the amount of mental effort (computational cost) required for the task. Among several types of computational costs, we focused on the effort of retrieving declarative knowledge. As described in Section 2.3.1, retrieval of declarative knowledge in ACT-R can be hypothesized to increase prefrontal cortex activity. Therefore, it can be reasonably assumed that deliberative levels of thinking, which affect the optimal level of intrinsic motivation (Csikszentmihalyi, 1990; Yerkes and Dodson, 1908), are estimated from the amount of declarative knowledge retrieved during the task execution.

**Figure 6:**
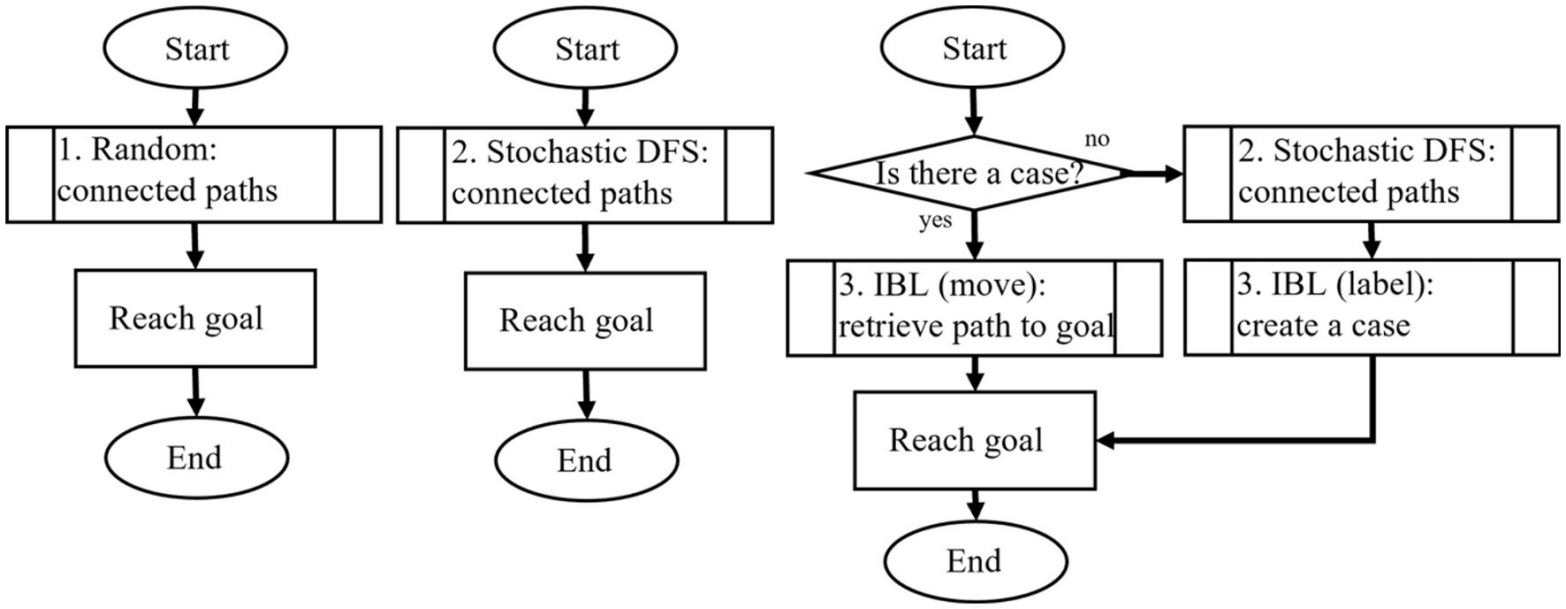
Manipulation of internal environments. DFS, depth-first search; IBL, instance-based learning.

Figure 6 depicts the manipulation of the levels of thinking in this study focusing on the maze search task. The process of the model became complex from left to right, and the amount of declarative knowledge used in the task was assumed to increase. These models were developed based on the authors' previous work (Nagashima et al., 2021) with two modifications; more complex pattern matching in the path retrieval and leveraging all pattern matching as triggers of intrinsic reward. In the previous research, the smallest number of variables in the productions was only one, so there was no pattern in the rule. Also, the previous research limited the triggers of intrinsic rewards only when the maze searching rules were fired, omitting rewards generated from pattern matching that occurred by other productions during the task.

These changes were made to ensure the model's consistency with our theoretical assumptions. There may be debate about assuming that every pattern match triggers intrinsic rewards. For example, it might be possible to prioritize pattern matching based on complexity or to select productions for positive rewards by setting certain criteria. However, in this study, we prioritized a simpler setting to verify the basic idea, avoiding any arbitrariness. The next sections explain the specific process of the model in each internal environment.

#### 4.3.1 Random model

The model with the lowest level of thinking randomly transitioned to the current location stored in the goal module. The model repeated the following process during each round until the goal was achieved or the time limit was reached.

1. Path search: the model retrieved the declarative knowledge related to the paths adjacent to the current location. To retrieve the declarative knowledge, the model used productions in which the current location was bound to a variable.
2. Move:
  (a) If the path retrieval was successful (pattern matching occurred), the model updated the state of the goal module according to the retrieved path, and the model returned to Step (1).
  (b) If the path retrieval failed, the model returned to Step (1) without modifying the state of the goal module.

While the model explored the maze using this search strategy, the productions that were used for the successful retrieval of the path were compiled. The model was assumed to have a few opportunities for pattern matching because production compilation occurred only when the stored path was retrieved. Therefore, stimulating intellectual curiosity in this model was considered as difficult.

#### 4.3.2 Stochastic Depth-first Search (DFS) model

**Figure 7:**
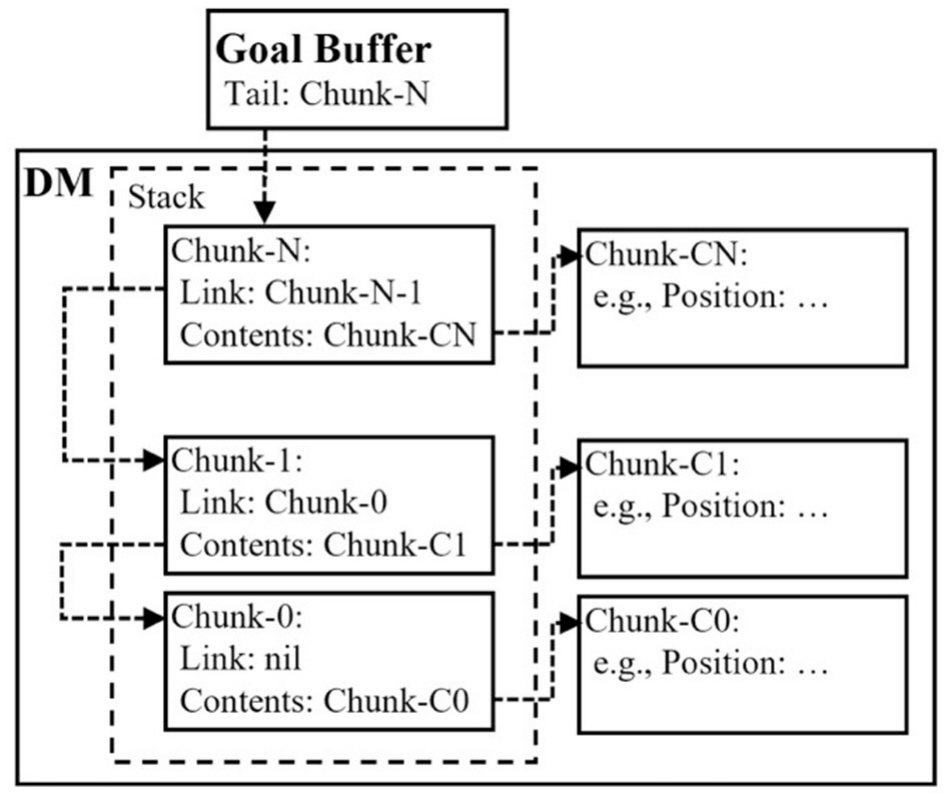
Construction of the stack structure using chunks of adaptive control of thought-rational (ACT-R) architecture. This stack was implemented using the imaginal module of ACT-R.

To include higher cognitive functions (declarative module), we constructed a probabilistic DFS model, which backtracked to search the environment based on the study by Reitter and Lebiere (2010). As indicated in Figure 7, the model exhibited a stacked structure with chunks generated by the imaginal module of ACT-R. The push function in the stack was realized by storing a chunk that contained the name of the previous chunk in the *Link* slot. Additionally, the pop function in the stack was realized by returning this slot value to the previous slot value. We implemented all these processes using only ACT-R productions without defining any external functions written in other programming languages, such as LISP.

Similar to the random model, the stochastic DFS model compiled productions that could retrieve declarative knowledge of paths and backtrack to learn new productions that did not contain variables. The specific model behavior can be summarized as follows.

1. Path search: the model determined the destination by retrieving the declarative knowledge associated with the path, similar to the random model. The IF clause included the current location stored in the goal buffer and five variables, corresponding to the current location and the directions (west, north, east, and south), which were flags indicating whether the direction was already searched or not.
2. Move:
  (a) If the knowledge retrieval was successful (pattern matching occurred), the model flagged the retrieved direction as “searched,” created a new chunk using the imaginal module, and stored the chunk as declarative memory, as depicted in Figure 7. Simultaneously, the model updated the current location of the goal buffer according to the retrieved path. At this point, the searched flag in the goal buffer was reset, whereas the searched flag in the direction opposite to the direction of movement was set to prevent its return to the previous location. After this procedure, the model returned to Step (1).
  (b) The backtracking process was executed if the path retrieval failed, returning the model to the previous state by popping chunks in the stack; eventually, the model returned to Step (1).

The model repeated this behavior until the goal was achieved or the time limit was reached. Contrary to the random model, the DFS model used the stack when the path search failed. Therefore, the model required more rounds to compress (compile the production) the declarative knowledge of the paths.

#### 4.3.3 Stochastic DFS plus Instance-based Learning (IBL) model

This model combined the stochastic DFS with the IBL, which leverages past memories to solve current tasks (Gonzalez et al., 2003; Lebiere et al., 2007). In this task, the model held all the retrieved paths in the stack from the beginning of each round until the goal was reached. After the model attained the goal, the path chunks in the stack were retrieved one by one, and the chunks labeled “correct path” were generated. During each round, the model repeated the following two steps until the goal was achieved or the time limit was reached.

1. Determining strategies: at the beginning of each round, the model decided between the stochastic DFS and the IBL strategies by retrieving chunks associated with the current location and labeled “correct path.”
2. Move:
  (a) When the DFS strategy was employed (failed to retrieve the “correct path”), the model behaved as a stochastic DFS model.
  (b) When the model successfully retrieved the “correct path,” the model updated the current location according to the retrieved path chunk. Subsequently, the model returned to Step (1).

The model behaved as the stochastic DFS model in the early stages of the task. With the repetition of rounds and the increase in the number of instances with the “correct path,” the model effectively reached the goal. Here, IBL was a time-consuming process in comparison with the DFS strategy. This was because the model had to retrieve the path in the stack at the end of the round to assign a label to a path. Moreover, retrieval trials for past successful rounds at the beginning of each round resulted in additional time, which was not included in the other models. We hypothesized that similar to the stochastic DFS model, this model is likely to stimulate intellectual curiosity, and the IBL function would positively affect the learning of the task.

#### 4.3.4 Deep reinforcement learning model based on curiosity

To explore the relationship between the aforementioned ACT-R models and previous models of intrinsic motivation using deep reinforcement learning, we constructed an ICM model based on the report by Pathak et al. (2017). The ICM model in this study explored the maze using the policy π in actor-critic model.^7^ This search resulted in a policy that maximized the rewards represented as


(4) $$\begin{array}{l} r_{t}=r_{t}^{i}+r_{t}^{e}. \end{array}$$

Thus, the reward of the model was calculated as the sum of the internal reward (*r*_*i*_) and external reward (*r*_*e*_). Based on this equation, the model explored the environment by balancing the two types of rewards.

In this study, following Pathak et al. (2017), the internal reward was determined by


(5) $$\begin{array}{l} r_{t}^{i}=\frac{\eta }{2}||\hat{\phi }\left(s_{t+1}\right)-\phi \left(s_{t+1}\right)|{|}_{2}^{2}. \end{array}$$

Here, state *s* is defined as pixel data in deep reinforcement learning. In this study, the maze situation (players, walls, and paths) was converted into a grayscale image (42 × 42) and served as input to a CNN, whose parameters were represented as ϕ. By subtracting the predicted and actual outputs of CNN, prediction errors were computed and weighted using the coefficient η. This coefficient was regarded as the intensity of curiosity.

By contrast, the external reward was defined as


(6) $$r_{e}=\{\begin{array}{ll} -1 & \text{if failed to move;}\\ 0 & \text{if succeeded to move;}\\ 10 & \text{if the goal was achieved.} \end{array}$$

In each action, the model attempted to select one of the directions, namely, west, north, east, or south, and to transition the state from one corner point to another. If the model selected a direction that did not lead to a path, the action was considered a failure. If the model reached the goal owing to its movement, it was rewarded for its success; subsequently, the task moved on to the next round.

The search for the maze was terminated when the condition


(7) $$\begin{array}{l} th<r_{i}\times 500+egs \end{array}$$

was satisfied. Here, *th* denotes the threshold value, and *egs* indicates noise. The model search was terminated when the internal reward was less than the threshold.^8^

### 4.4 Simulation settings

#### 4.4.1 Setting for ACT-R models

As parameters relevant to the general model of task continuation (Figure 4), the simulation assigned the initial utility values of the *continue* and *stop productions* to 10 and 5, respectively. Additionally, we assigned the triggers of the negative reward (*r* = 0) to productions that recognized the end of the round, which was either reaching the goal or recognizing that the time limit of each round was elapsed. Conversely, the triggers of the positive reward were assigned to productions that included pattern matching, which corresponded to intellectual curiosity. We manipulated the intensity of the model's intellectual curiosity by sampling the positive reward at five equal intervals, ranging from 2 to 18. For parameters not directly related to our proposed mechanism, we adopted values from previous studies. Following Anderson et al. (2004), the activation noise level (ANS), which represents the noise in memory recall, was set to 0.4, and the production noise level (EGS), which reflects the noise in comparing utilities for continuing or terminating productions, was set to 0.5.

To enable the above setting of rewarding by pattern matching, we made small modifications to the original source code of ACT-R (Ver. 7.21). We first modified the source code of ACT-R to assign the reward trigger at any time point after the production compilation occurred. Subsequently, we modified the code to not inherit those triggers after the compilation. In the original ACT-R source code, the compiled production inherits the reward triggers from the original production. We redefined this function to represent boredom caused by the lack of new production compilation.

Simulations based on these settings were run 10 times for each map and each positive reward setting. The limits in the ACT-R simulation time for each round and run were set to 180 and 3,600 s, respectively.

#### 4.4.2 Setting for ICM model

The ICM model was implemented using PyTorch (ver. 1.9.0), with parameters set to match those of the ACT-R, wherein the simulations were run on 30 maps and the proportion of the internal reward (η) for each run was divided into five samples with equal intervals, ranging from 0.1 to 0.9. We compared the sum of the internal reward (*r*_*i*_) and the noise (*egs*) with the threshold (*th* = 5) in Equation 7 to determine whether the task was continued or terminated. Furthermore, we set 156 and 3,130 steps as the limit of the action in each round and run, respectively. These steps were set to be equivalent to the time limit set at the ACT-R models. One step of the ICM model was equivalent to the rule transition time of 1.15 s in the default random model. The ICM model was run 100 times for each reward setting as it could run faster than the ACT-R models.

### 4.5 Simulation results

**Figure 8:**
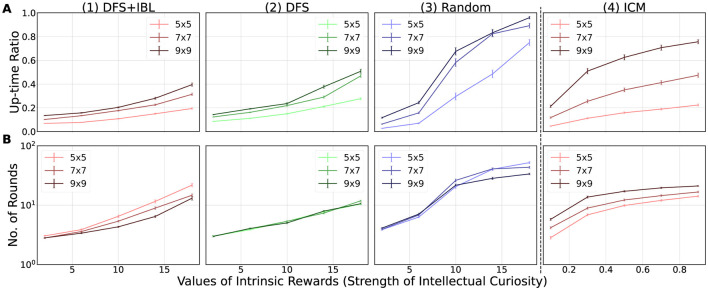
Simulation results. The numbers in the horizontal line distinguish the models (1–3: adaptive control of thought-rational (ACT-R) models; 4: intrinsic curiosity module (ICM) model), and the vertical alphabet differentiates the indicators (**A**: Up-time ratio; **B**: Number of rounds). The error bars in each graph indicate the mean value (*n* = 10) of the standard deviations (ACT-R: *n* = 10, ICM: *n* = 100) obtained for each map when multiplied by 1/10.

**Figure 9:**
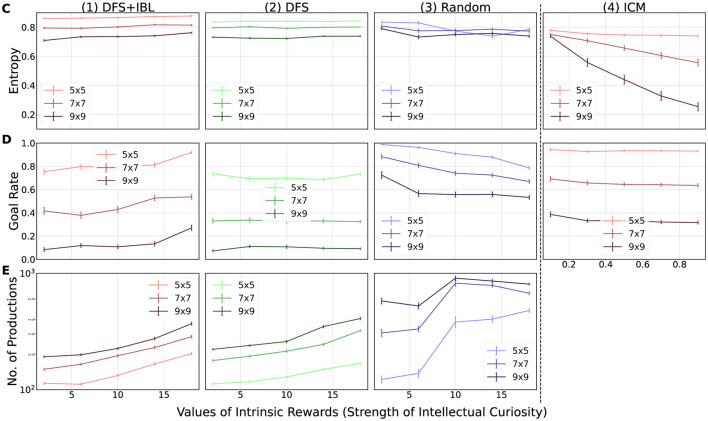
Simulation results. The numbers in the horizontal line distinguish the models (1–3: adaptive control of thought-rational (ACT-R) models; 4: intrinsic curiosity module (ICM) model), and the vertical alphabet differentiates the indicators (**C**: Entropy; **D**: Goal rate; **E**: Number of productions). The error bars in each graph indicate the mean value (*n* = 10) of the standard deviations (ACT-R: *n* = 10, ICM: *n* = 100) obtained for each map when multiplied by 1/10.

Figures 8, 9 illustrate the simulation results as a function of the internal reward for each of the indicators discussed in Section 4.1. Each graph depicts the average value, which was *n* = 100 (10 times × 10 maps) for the ACT-R models and *n* = 1, 000 (100 times × 10 maps) for the ICM model, aggregated for each internal and external environment condition with respect to the map size. The influence of the maps of the external environment was examined by comparing the three series in each graph, whereas the influence of the internal environment (random, DFS, DFS + IBL) of the model was analyzed based on the difference between the graphs aligned in the horizontal direction. The subsequent sections discuss the obtained results based on the three questions posed as objectives of the simulation.

#### 4.5.1 Environment that stimulates intellectual curiosity

In Section 4.1, the first question posed was “What type of environment stimulates intellectual curiosity?” To address this question, we focused on the up-time ratio (Figure 8A) and number of rounds (Figure 8B) as the behavior indicators of intellectual curiosity. These indicators increased continuously with the increase in the strength of intellectual curiosity for every series (map size) in every graph (levels of thinking) of Figure 8. This general trend suggested that the implemented intellectual curiosity actually enhanced the motivation for the task and ensured task continuation.

In terms of the difference in the external environment, the up-time ratio (Figure 8A) increased as the map became more complex (9 × 9>7 × 7>5 × 5). However, the number of rounds (Figure 8B) presented a reverse trend, wherein the simpler external environment increased the number of rounds (9 × 9 < 7 × 7 < 5 × 5), except for the DFS model (Figure 8B2). The discrepancy between the two indices of motivation was caused by the time limit of the simulation (3,600 s). The model could complete the simple map faster, resulting in a greater number of rounds within the time limit (Figure 8B). However, as indicated by Figure 8A, the simple map enabled the model to terminate the task early owing to the lack of new patterns for production compilation. This result implies that the complex external environment stimulates intellectual curiosity.

Furthermore, we determined the difference between the internal environment, which was not expected in advance. When comparing the three horizontally aligned ACT-R models, we observed that the models with high levels of thinking (DFS + IBL and DFS) had smaller indicators of motivation than the random model. The reason for this difference could be the advantage of the random model with less thinking time and more trials and errors. In this condition without physical constraints, the random model had a better chance of receiving positive rewards by identifying novel paths than the other models.

#### 4.5.2 Effect of task continuation on model learning

The second question posed was “How does stimulated intellectual curiosity affect task learning?” Figure 9 presents the three learning indices, namely, the changes in behavior (Figure 9C: entropy), the learning outcome (Figure 9D: goal rate), and the changes in internal state (Figure 9E: the number of productions).

Based on the analysis of Figure 8, we confirmed that all conditions of the internal and external environments were stimulated by intellectual curiosity. However, Figure 9 indicates that the effect of intellectual curiosity on task learning differs depending on the internal environment. The intensity of intellectual curiosity affected positively for higher levels of thinking. In the highest level of thinking (the DFS + IBL model), all learning indices (Figures 9C1, D1, E1) increased with the intensity of intellectual curiosity. In the middle level (the DFS model) increased the number of productions (Figure 9C2) while maintaining the entropy (Figure 9C2) and goal rate (Figure 9D2). In the case of the lowest level (the random model), the intrinsic motivation decreased all indices (Figures 9C3, D3, E3). These trends indicated that the DFS + IBL model exhibited a goal-oriented behavior because of the learning effect of the IBL strategy, whereas the behavior of the DFS model had to search the entire map. Furthermore, the random model did not lead to the goal; this was because the model reinforced unfavorable behavior by repeatedly visiting the same location without expanding the search.

In terms of the effect of the challenge of the task (task difficulty), the entropy (Figure 9C) and the goal rate (Figure 9D) were greater on the small map, whereas the number of productions was higher on the large map. These differences may be attributed to the fact that the small map was easier to explore, which in turn increased the entropy and the goal rate. Conversely, the large map exhibited more pattern-matching opportunities, leading to more accumulated knowledge by frequent compilation.

In summary, intellectual curiosity promoted learning in the DFS + IBL model, which exhibited the highest level of thinking. By contrast, learning in the DFS and random models was not promoted by intellectual curiosity. Moreover, the effect of intellectual curiosity negatively impacted the learning environment in the random model.

#### 4.5.3 ACT-R curiosity vs. ICM curiosity

Finally, we compared the ICM and ACT-R models in Figures 8, 9. Similar to all ACT-R models, the ICM model was stimulated by stronger intellectual curiosity (Figure 8). However, the effect of intellectual curiosity for task learning was specifically similar to the random ACT-R model that exhibited decreasing trends of the entropy (Figure 9C4) and the goal rate (Figure 9D4) with the increase in the strength of intellectual curiosity. With respect to the effect of the external environment, the ICM model was also similar to the random ACT-R model; the up-time ratio (Figure 8A4) and the number of rounds (Figure 8B4) were greater for larger maps, whereas the entropy (Figure 9C4) and the goal rate (Figure 9D4) were greater for smaller maps.

This comparison confirmed commonalities and differences between the developed ACT-R curiosity model and the existing curiosity model in deep reinforcement learning. The proposed ACT-R curiosity mechanism can represent similar learning to the existing model by including a simple internal environment (random search strategy). At the same time, it can incorporate goal-directed behavior by including “explicit use of success memory.” Such an explicit nature of the proposed mechanism also leads to a direct examination of the model's internal learning. The analysis of Figure 9E clearly shows this advantage of interpretability made by the proposed approach.

#### 4.5.4 Cases of paths discovered by the models

**Figure 10:**
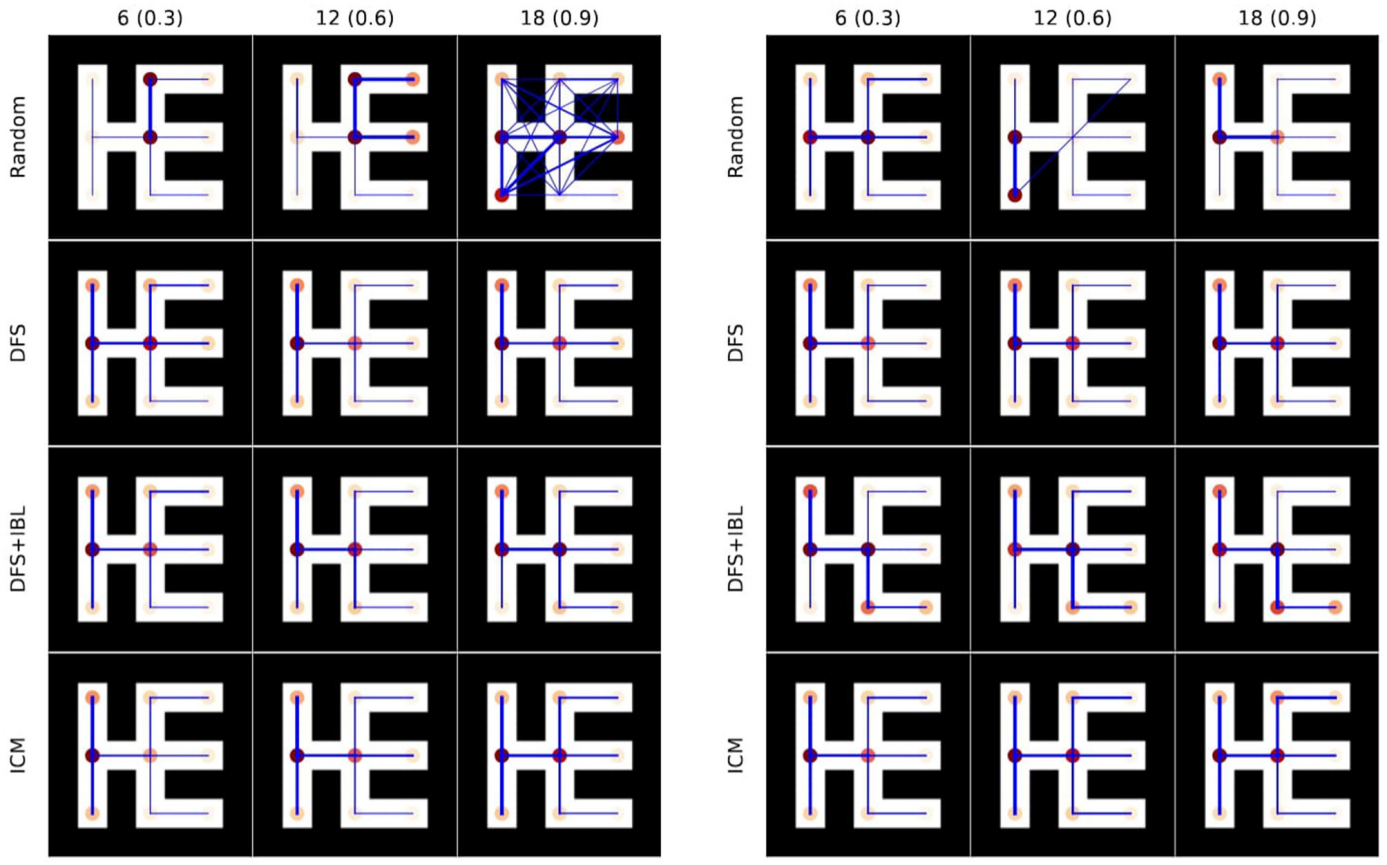
Trajectories of the model runs [**(left)**: low-performance runs, **(right)**: high-performance runs]. The columns indicate the strength of intellectual curiosity, while the rows correspond to each model.

To compare detailed behaviors between models, Figure 10 illustrates example paths in a 5 × 5 map. The map depicts start and goal positions at the top left and bottom right corners respectively. The circles' colors and line thickness represent visit frequencies during runs. The random model exhibited diagonal movement and movement through walls, a result of compiling multiple movement rules. To gather these examples, we conducted 10 runs for each model across three levels of intrinsic rewards, selecting the runs with the lowest and highest performance for analysis.

These figures reveal distinct behavioral characteristics of each model. The random model predominantly exhibits localized movements within specific areas, often distant from the goal. On the other hand, the DFS model explores the map evenly but does not necessarily move directly toward the goal. In contrast, the DFS + IBL model demonstrates deliberate behaviors aimed at reaching the goal, particularly under high-reward conditions. In terms of localized movement patterns, the ICM model was more similar to the random and DFS models than the DFS + IBL model. Thus, the results suggested that the DFS + IBL model had a greater effect on curiosity strength than the other models regarding directionality toward the goal. These observations support the findings observed in the quantitative results shown in Figures 8, 9.

## 5 Conclusions

The objective of this study was to develop a mechanism for intrinsic motivation based on pattern discovery by combining basic modules of ACT-R. This section summarizes the significance of the proposed mechanism and presents the potential future lines of investigation.

### 5.1 Summary and implications

The proposed mechanism was based on the assumption that pattern discovery is associated with the feeling of fun and is the source of intellectual curiosity. Additionally, its attenuation was expressed by the learning mechanism incorporated in ACT-R. To support this proposal, we implemented multiple external environments (challenges in the task) and strategies for exploring the external environment (levels of thinking) and examined the role of intellectual curiosity in each situation. The simulation results indicated that the rewards associated with pattern discovery exhibited different effects on models at different levels of thinking. The model with the lowest level of thinking (random) and that with the middle level of thinking (DFS) had negative and neutral effects of intellectual curiosity on performance, respectively. The only model that benefited from intellectual curiosity was the one with the highest level of thinking (DFS + IBL), which comprised a function that enabled it to remember previous experiences that led to the goal.

These results are partially consistent with the past arguments made for human intrinsic motivation. Particularly, the effectiveness of intrinsic motivation in the DFS + IBL model is consistent with a discussion, in which intrinsic motivation operates well with deliberative thinking, which requires “autonomy,” “mastery,” and “purpose” (Pink, 2011). Furthermore, consistent with our negative results in the random model, several reports exist on behavioral addictions caused by the negative effects of intrinsic motivation (Alter, 2017). For instance, people often forget their goals and become engrossed in exploratory tasks, such as browsing the internet, resulting in poor performance. This irrational behavior might also relate to *computational psychiatry* (Huys et al., 2016).

In addition to the above discussion on the internal environment, we identified a connection with a previous discussion on the external environment. Consistent with the discussion on “challenge” (Malone, 1981), we determined that the up-time ratio in larger maps was greater than that in the smaller maps. This result indicates that a complex external environment stimulates intellectual curiosity. However, we also determined that difficult challenges generate ineffective learning on a wide map (Figure 9). The aforementioned positive and negative effects of the task difficulty indicate the optimal level of challenge (Csikszentmihalyi, 1990; Yerkes and Dodson, 1908).

Furthermore, this study successfully corresponded with past studies on intrinsic motivation in reinforcement learning. The comparisons with the ICM model (Pathak et al., 2017) confirmed that the developed ACT-R model, specifically the random model, is a succession of existing studies. Although we cannot claim its superiority as a learning algorithm based on the current simulation alone, the model with the higher level of thinking (DFS + IBL) exhibited characteristic behavior toward the ICM model. Future analysis of more extensively manipulating parameters, such as the balancing of *r*_*i*_ and *r*_*e*_ in Equation 4 and designing the external environment stimulating curiosity (Burda et al., 2018), could reveal further correspondence between the ACT-R model and reinforcement learning framework.

We believe that the comparisons of the previous model of reinforcement learning reveal the methodological advantage of using cognitive architecture. An integrated cognitive architecture, such as ACT-R, provides criteria to set numerical parameters (e.g., time limits and utilities) based on previous studies. Furthermore, ACT-R comprises neuroscientific modules that correspond to basic cognitive functions, such as declarative and procedural knowledge. Based on this relation, arguments associated with human intrinsic motivation can be developed. Therefore, this study contributes to the understanding of intrinsic motivation in a wide context of the relationship between human evolution and the development of civilization by mapping the discovery of patterns to intrinsic motivation (Baron-Cohen, 2020).

### 5.2 Future work

The proposed mechanism of intellectual curiosity has the potential for several future studies. The primary focus among them is human experiments that manipulate the internal and external environments as in the simulation. A simulation study without data is nothing more than a demonstration derived deductively from theory. Therefore, the model's value must be proven by applying it to human scenarios.

One of the obstacles to conducting human experiments for the proposed mechanism is setting tasks to stimulate human curiosity. In this study, we adopted the maze task because several previous researchers based on ACT-R have constructed models for this task. However, setting experimental situations with human participants to exhibit intrinsic motivation for solving such simple tasks may be difficult. Therefore, in the future, we intend to explore tasks that both humans and developed models can execute with proper intrinsic motivation.

Other future work will focus on modeling the curiosity and motivation that was not explained in the current study. As we discussed in Section 3.1, this study targeted on intellectual curiosity relating “a desire to bring better form to one's knowledge structures” (Malone, 1981) or “intrinsic desire to build a better model for the world” (Schmidhuber, 2010). Therefore, we have not yet explained the sensory curiosity that drives us to acquire new knowledge from the world. These two types of curiosity are considered complementary, similar to the explore-exploit trade-off in reinforcement learning. Without including sensory curiosity in the model, we cannot explain how declarative knowledge is acquired for intellectual curiosity, nor how the initial utility settings of continuing the task exceed those of stopping.

The above future study possibly leads to a deeper exploration of levels of thinking. Conway-Smith et al. (2023) recently summarized the relationship between metacognition and levels of thinking, arguing that compilation of existing knowledge reduces the effort involved in metacognition, making it more automatic. Building on this, we can suggest that achieving such a metacognitive state as a result of higher-level thinking enabled with enough intellectual curiosity, exemplified by the DFS + IBL model.

On the contrary, we can assume the exploratory role of the lower level of thinking. As shown in Figure 9, the random model showed a higher goal ratio with larger learning products in some conditions. These results suggest links between low-level thinking and sensory curiosity, leading to exploration of the environment. Our recent work (Nagashima and Morita, 2024) provides support for the above interpretation. In the experiment, human participants observed the behaviors generated by the models in the current study and rated the random model as having the most curious features.

The final direction for future research is the generalization of the ideas presented in this paper to other tasks in real-world settings. We believe such tasks are linked to the earlier discussion on the civilization of society (Baron-Cohen, 2020). As suggested by Toya and Hashimoto (2018), tool-making requires recursive compilation of intermediate products. Integrating this multi-agent simulation with the mechanisms proposed in the current study could offer a detailed explanation of the driving forces behind the evolution of civilization.

## Data Availability

The original contributions presented in the study are included in the article/Supplementary material, further inquiries can be directed to the corresponding author.

## Footnotes

^1^The mission of the society for affective science includes “motivated states.” See https://society-for-affective-science.org/about-sas/.

^2^Claimed by Anderson (2007) in the chapter featuring “dynamic pattern matching” in ACT-R.

^3^A similar mechanism of stopping a navigation task was presented by Anderson et al. (1993), although the exact equations of utility calculation were different because of the architectural difference.

^4^The models and data are included in https://github.com/AcmlNagashima/CuriosityAgents.

^5^https://algoful.com/Archive/Algorithm/MazeExtend.

^6^The exclusion of perceptual and motor processes in basic simulations is also recommended in the official ACT-R tutorial.

^7^Discount rate *gamma* = 0.99.

^8^A fixed value of 500 was tentatively multiplied because the scales of the internal reward in the ACT-R and ICM models were different.
